# Exploration and Exploitation Approaches Based on Generative Machine Learning to Identify Potent Small Molecule Inhibitors of α-Synuclein Secondary Nucleation

**DOI:** 10.1021/acs.jctc.2c01303

**Published:** 2023-03-20

**Authors:** Robert I. Horne, Mhd Hussein Murtada, Donghui Huo, Z. Faidon Brotzakis, Rebecca C. Gregory, Andrea Possenti, Sean Chia, Michele Vendruscolo

**Affiliations:** † Centre for Misfolding Diseases, Yusuf Hamied Department of Chemistry, University of Cambridge, Cambridge CB2 1EW, United Kingdom; ‡ College of Life Science and Technology, Beijing University of Chemical Technology, Beijing 100029, China; § Bioprocessing Technology Institute, Agency of Science, Technology and Research (A*STAR), Singapore 138668, Singapore

## Abstract

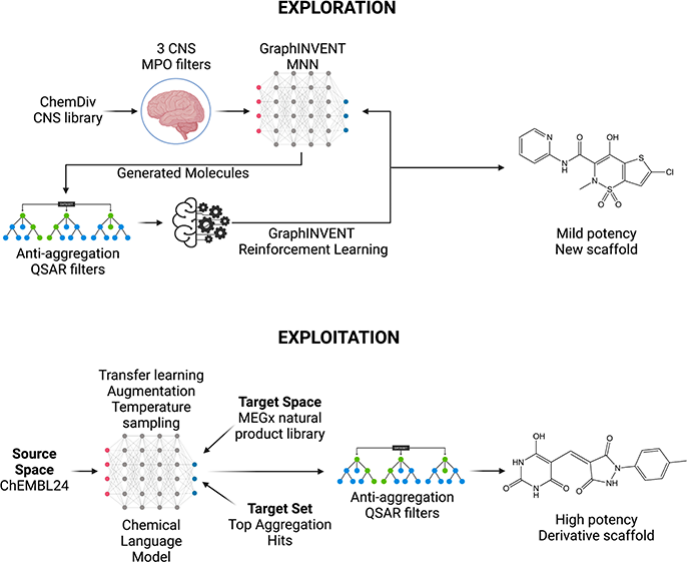

The high attrition rate in drug discovery pipelines is an especially pressing issue for Parkinson’s disease, for which no disease-modifying drugs have yet been approved. Numerous clinical trials targeting α-synuclein aggregation have failed, at least in part due to the challenges in identifying potent compounds in preclinical investigations. To address this problem, we present a machine learning approach that combines generative modeling and reinforcement learning to identify small molecules that perturb the kinetics of aggregation in a manner that reduces the production of oligomeric species. Training data were obtained by an assay reporting on the degree of inhibition of secondary nucleation, which is the most important mechanism of α-synuclein oligomer production. This approach resulted in the identification of small molecules with high potency against secondary nucleation.

## Introduction

A link between α-synuclein (αS) aggregation and Parkinson’s disease (PD) is supported by genetic evidence and by observations of the accumulation of αS in inclusions known as Lewy bodies in the brains of PD patients.^1−3^ A primary aim of current research toward the development of therapeutic treatments of this disease is therefore the inhibition of αS aggregate formation.

Our approach here is based on the realization that it is particularly important to target αS aggregation by specifically preventing the formation of αS oligomers.^4^ These intermediate species are particularly cytotoxic, as they can disrupt cell membranes, especially those of mitochondria.^5−7^ In order to reduce the number of oligomers produced in an aggregation reaction, one should take into account that highly ordered fibrillar aggregates can act as highly effective catalytic surfaces for oligomer formation.^8^ The pathological relevance of these processes has led to major investment into identifying compounds that can inhibit those aggregation mechanisms associated with neurotoxicity.^9−12^ As therapies are beginning to be delivered for Alzheimer’s disease,^13^ the race is on to achieve the same outcome for PD patients.^14−16^

Computational methods can contribute to these endeavors. In particular, in recent years, deep learning has emerged as a powerful tool for cheminformatics.^17^ With this capability, molecular generative models have emerged as promising tools for de novo molecular design. It has also been previously shown that computational methods can offer more efficient routes to αS aggregation inhibitors than traditional screening methods.^18,19^ A limitation of that approach was the use of pre-existing screening libraries, which biased the model and limited the search space. A further limitation was focusing only on the molecule potency during the machine learning task.

The present work aims at addressing these shortcomings through the application of generative modeling approaches and multiparameter optimization in two separate pipelines: (1) one focused on *exploration* (identifying novel and effective molecular structures), and (2) the other on *exploitation* (achieving higher potency from known chemical space). The former employs an architecture derived from the GraphINVENT^20^ framework for multiparameter generative modeling while the latter consists of a chemical language model optimized for low data regimes.^21^

Both pipelines feature a generative model linked to a QSAR (quantitative structure activity relationship) filter. QSAR models are incorporated into generative pipelines to enable learning of the underlying relationship between the molecular structure and activity in silico.^22^ Consequently, a smaller number of candidate molecules need to be tested in vitro. However, many constraints are involved in QSAR model training, such as the high dimensionality and sparsity of molecular fingerprints, in addition to the high correlation of the chemical descriptors. This makes ensemble learning models, especially Random Forest models (RFs), which are convenient and robust for this task.^23^ Moreover, one great advantage of RFs is interpretability, meaning they can be beneficial in identifying the common features of molecules with high activity levels against the target. The QSAR models in this project predict whether a molecule can delay αS secondary nucleation. As the experimental aggregation inhibition data set produced previously^19^ was small (453 molecules) and imbalanced, we made efforts to train several QSAR models to maximize accuracy.

### Exploration

In the initial phase of the exploration pipeline, a graph-based generative model was trained to generate drug-like molecules that could penetrate the blood–brain barrier (BBB) and reach the central nervous system (CNS). Then, the generative model was fine-tuned using reinforcement learning to generate the molecules with other desired properties, including potency. To implement this plan, we defined a scoring function based on two complementary QSAR molecular activity classifiers trained on experimental data. Since RFs make predictions by combining the results of a set of individual decision trees that train simultaneously on subsets of the data set,^24^ the number of predictors and their correlations do not create problems for RFs. These models were used in the reward function of a reinforcement learning model to generate new molecules with the desired activity. Using this architecture, we generated small molecules predicted to penetrate the BBB, and potentially delay αS aggregation.

Most of the molecules, while synthetically accessible, were unavailable from screening libraries without custom synthesis at high expense. While the systematic experimental testing of these molecules was not possible for this reason, they showed strong overlap in the chemical space with active hits reported previously,^18,19^ providing support for their intended effect. In one case, a molecule was available, and was tested from the generative model training set, which had been used in transfer learning to allow the model to create valid molecular structures. This molecule was predicted by the QSAR filters to have strong CNS properties and a good antiaggregation score, and it showed mild inhibition in the same range as the existing clinical aggregation inhibitor Anle-138b.^12^

### Exploitation

An exploitation strategy had been previously reported,^19^ which required use of a restricted area of the chemical space, as it involved screening a library of available compounds with a degree of similarity to the initial hits. We sought here to remedy this limitation via the use of a generative chemical language model^21^ (CLM), designed to function in the low data regime of this project and trained on the same aggregation set as used in the exploration pipeline. This approach employed: (i) transfer learning, (ii) temperature sampling, and (iii) data augmentation to enable the model to ably construct valid molecules with applications to the area of interest, despite very few data points. For transfer learning, the model was pretrained on a synthetic compound space of bioactive molecules (ChEMBL24) to enable it to construct valid molecules with an increased likelihood of bioactivity. The model also uses a library of natural products (MEGx collection, Analyticon Discovery GmbH) as a target space to optimize toward, thus indirectly optimizing the pharmacokinetics of the resultant compounds via incorporation of features of a bioactive library, rather than using a parameter such as CNS MPO as above. Temperature sampling and data augmentation via shuffling of SMILES strings ensured the model achieved uniqueness, validity, and novelty. As with the exploration pipeline the resulting molecules were screened for potency, yielding hits that rivalled the best molecules from the previous exploitation model in terms of potency.^19^ These molecules far outstripped the hit found via the GraphINVENT approach in terms of potency, which illustrates the greater challenge presented by explorative scaffold hopping compared to exploitation of known chemical space. The weaker hit could nonetheless be optimized via the exploitative approach described here, in a synergistic strategy combining the exploration and exploitation pipelines in series.

## Results

### Exploration Pipeline

#### Creation of a Library of Small Molecules with Good CNS Penetrance

To compile the training data set, we curated the CNS drug libraries of small molecules provided by ChemDiv.^25,26^ In addition to these libraries, we added the molecules provided by the B3DB^27^ data set, a benchmarking data set of BBB molecules compiled from 50 published resources and removed duplicates, creating a data set of 37,895 molecules. The data set was further filtered and assessed by a BBB permeability binary classifier,^28^ pretrained on experimental brain permeability data, a CNS MPO score predictor,^29^ and a CNS MPO score calculator.^30^

**Figure 1:**
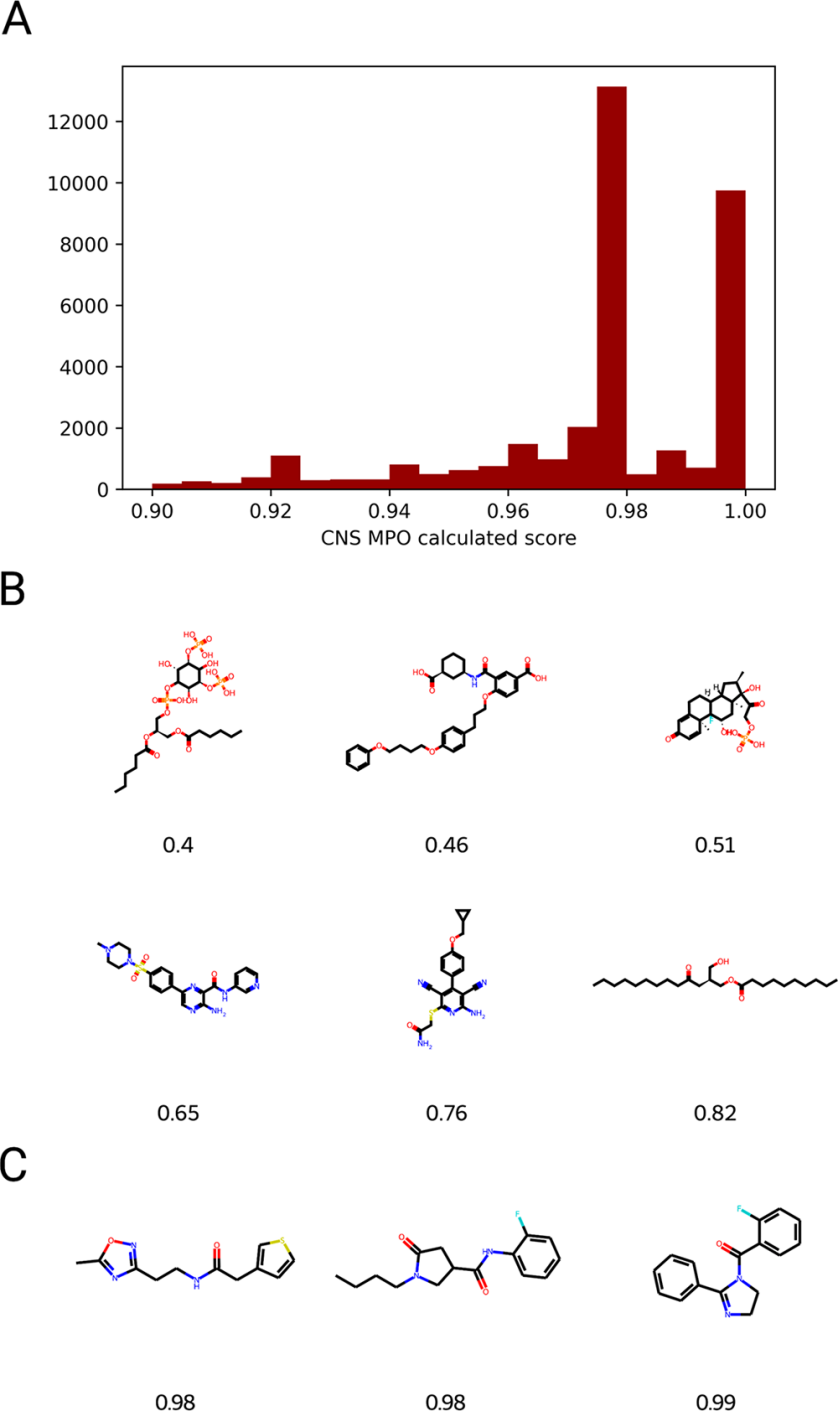
Creation of a library of small molecules with good CNS penetrance. (A) Calculated CNS MPO scores (GuacaMol) for the library subset of 35,636 molecules after filtration through the 3 different scoring methods (see text). (B) Randomly selected molecules spanning a range of lower CNS MPO values. (C) Representative molecules from the filtered set are shown. This data set was then used as the training set for molecule generation.

The CNS MPO scores are a commonly used metric for BBB penetrance in drug discovery and medicinal chemistry.^30,31^ However, it is not possible to obtain the CNS MPO score of a molecule without using a machine learning predictor, given that the p*K*_a_ value cannot be readily calculated from the structure of a molecule, unlike other properties.^32^ This makes the CNS MPO score prediction a regression task that highly depends on the precision of the p*K*_a_ prediction. Therefore, in this project, we used multiple CNS MPO predictors to filter the initial library. A BBB permeability binary classifier (DeePred-BBB,^28^ using PaDEL^33^ molecular descriptors as input features) and a CNS MPO score calculator not incorporating p*K*_a_ prediction (GuacaMol^29^) were used alongside another CNS MPO calculator^30^ that did incorporate p*K*_a_ prediction. The first classified a molecule as penetrant or not using a database of experimentally tested molecules. This model had high precision and AUC scores (0.98 and 0.99, respectively) and good generalizability in the original work. The second filter calculated a probability between 0 and 1 for relevant molecular properties of the molecule used in the CNS MPO score (molecules that achieved >0.9 on average passed the filter). The third filter^30^ again scored 6 calculated or predicted molecular properties (including p*K*_a_) between 0 and 1, and any molecule scoring a summed score of >4 was considered to pass. Eventually, after filtering, the data set only contained the molecules that were classified to be BBB permeable by all 3 filters, removing 2260 molecules from the initial CNS ChemDiv data set. The distribution of CNS MPO scores calculated via GuacaMol for the filtered set and the structures of representative molecules within that set are shown in Figure 1, alongside structures with a range of lower CNS MPO values for comparison. This filtered data set became the training set for subsequent generative modeling.

#### Generative Modeling

The GraphINVENT architecture was employed to generate molecules with desired properties. To convert the SMILES strings of the filtered CNS data set to graphs, each SMILES string was turned into a node feature matrix, an adjacency tensor, and a vector *r* that resembles a step-by-step decoding route for the molecule, i.e. steps to build the molecule starting from an empty graph. To obtain the vector *r*, the first step was the fragmentation of the molecular graph in a stepwise fashion using an algorithm developed in GraphINVENT. On each iteration, one edge/node was removed from the molecular graph G, and an action probability distribution (APD) was calculated for the new graph *G*_*n–1*_ until an empty graph was reached. Eventually, by aggregating APDs for all subgraphs *G*_*n*_, *G*_*n–1*_, *G*_*n–2*_, ..., we obtained the vector *r*
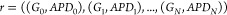
1

The removal order of nodes and edges of the graph is determined by a breadth-first search (BFS) traversal.^34^

We trained models to generate BBB-penetrant molecules and monitored the performance of the model in this respect. Therefore, we added three more evaluation metrics to GraphINVENT, using the 3 filters mentioned earlier: (1) the fraction of BBB permeable molecules, (2) the average calculated CNS MPO score, and (3) the average predicted CNS MPO score. These metrics were calculated for the novel molecules set generated by the model while training every 2 epochs. To calculate the BBB permeable molecule fractions, the chemical descriptors of the generated molecules were computed using the PaDEL software.

We selected the two top performing models from the GraphINVENT package, the gated graph neural network (GGNN) and the message neural network (MNN) and trained them with learning rates of 1 × 10^–4^ and 1 × 10^–5^ (4 training tasks in total). For each task, we split the data set into 80% training and 20% validation and trained the model for 100 epochs. The MNN was found to run more efficiently given its less complex message passing and aggregation functions.

The training was done in mini batches of 50 molecules, with a block size of 1000 molecules. As loss function, we used the Kullback–Leibler^35^ divergence, which measures the difference between probability distributions. In our case, the probability distributions to be compared are the target APD (*P*) and the predicted APD (*Q*) as
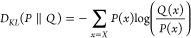
2

An Adam optimizer was used with weight decay (L2 regularizer). The model was used to generate a batch of 100 new molecules every 2 epochs. These molecules were evaluated using the original GraphINVENT scoring metrics (Table 1) and the BBB permeability and CNS MPO metrics (Tables 1 and 2, respectively) implemented above. The goal was to determine the best combination of model architectures and learning rates, in addition to the epoch number in which the model performed best.

**Table 1:** Metrics of Molecules Generated by MNN and GGNN at Their Best Performing Epoch for 2 Different Learning Rates^a^

	Metric^b^			
Model (learning rate)	Epoch	BBB Fraction	Valid Fraction	Unique Fraction
GGNN (1 × 10^–4^)	72	1.0	0.92	1.0
GGNN (l × l0^–5^)	66	1.0	0.94	1.0
MNN (l × l0^–4^)	90	1.0	1.0	1.0
MNN (l × l0^–5^)	84	0.925	0.84	1.0

a The BBB fraction is the fraction of molecules classified as brain penetrant by DeePred-BBB.

b Metrics are reported for the optimally performing epoch.

**Table 2:** CNS MPO Average Score Comparison at the Same Epochs as in Table 1^a^

	Metric^b^				
Model (learning rate)	Epoch	Calc. CNS MPO	Change from original	Predicted CNS MPO	Change from original
GGNN (l × l0^–4^)	72	0.964	–0.011	5.090	–0.170
GGNN (l × l0^–5^)	66	0.963	–0.012	5.195	–0.075
MNN (l × l0^–4^)	90	0.973	–0.002	5.337	+0.077
MNN (l × l0^–5^)	84	0.972	–0.003	5.330	+0.070

a The calculated CNS MPO score ranges between 0 and 1, 1 implying a very high probability of BBB penetrance, while the predicted CNS MPO score ranges between 0 and 6.

b Metrics are reported for the optimally performing epoch. Each metric has an associated “Change from original” column, which refers to the mean change between the generated population and training set.

**Figure 2:**
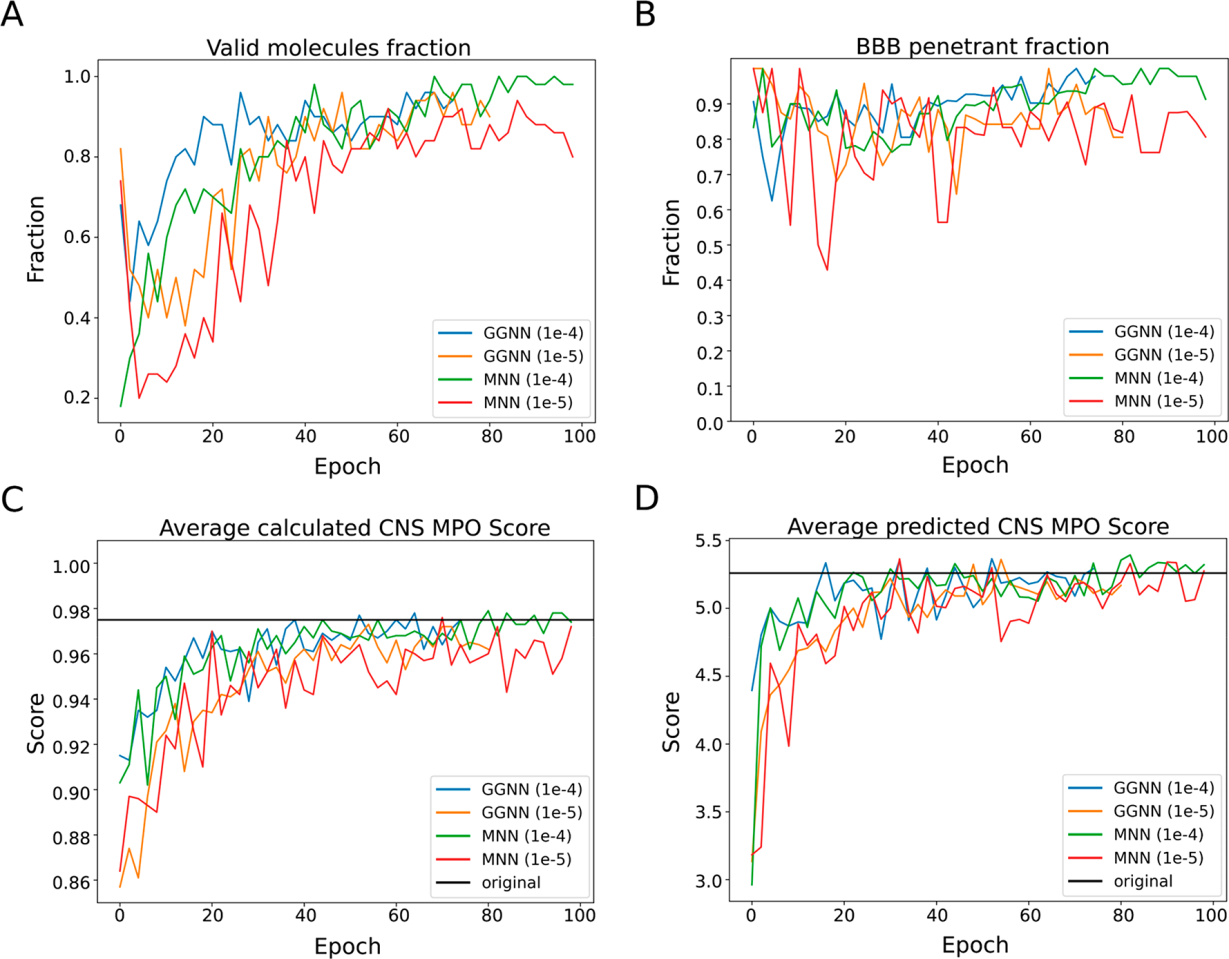
Metrics of generated small molecules during training with the GraphINVENT Gated Graph Neural Network (GGNN) and Message Neural Network (MNN) using 2 different learning rates. (A) Fraction of chemically valid molecules at each epoch. (B) Fraction of molecules passing the DeePred-BBB permeability classifier at each epoch. (C) Average calculated CNS MPO score using the GuacaMol implementation at each epoch. (D) Average predicted CNS MPO scores obtained using a previously reported method^31^ at each epoch (black line indicates average of the filtered training set).

We observed that the MNN (1 × 10^–4^) model outperformed the other three conditions in all metrics (Table 1 and Figure 2). All generated molecules were valid, unique, and BBB permeable. Moreover, the average predicted and calculated CNS MPO scores of its generated molecules were the closest to the score averages of the training data. Hence, this model was selected to be fine-tuned via reinforcement learning.

#### Reinforcement Learning SMILES Embedding-Based Reward Function

Having created a generator of BBB penetrant molecules, we now focused on tailoring these molecules for potency against αS aggregation. Limitations were the size of the data set available for this task, consisting of 453 molecules, and the unbalanced nature of the data set (Figure S1), making the development of a high performing model challenging. In this initial proof-of-principle study, we employed transfer learning to at least in part remedy the data set size limitation. As a further measure, data were oversampled to ensure data set balance. The applied oversampling was a simple data augmentation by random duplication of the active molecules. Data were scaled afterward and split into training and testing sets (80%–20%). The metric of potency was the normalized half time (*t*_*1/2*_) of aggregation, i.e. the time point at which 50% of the monomeric protein had aggregated, divided by the same 50% time point for the negative control. A hit was classified as any molecule with a normalized half time of 1.5 or greater. We note that none of the active molecules in the aggregation data set were present in the generative model training set, as we were aiming to identify novel structures.

For transfer learning a pretrained mol2vec skip-gram model trained on a diversified set of 19.9 million molecules was used, so that the QSAR model would not have to learn molecular representations from scratch. The first hidden layer of the network was a frozen embedding layer initialized with the weights of the mol2vec model (these were preserved throughout training). The output of this layer was a 2D embedding vector generated based on the weights from the base model. The next three layers were convolutional layers with a kernel size of 10 and a ReLU activation function. Between these layers, max pooling and dropout layers were added to reduce overfitting and minimize the feature space, followed by an LSTM layer that greatly improved the performance, given its ability to identify trends in the data. Lastly, two dense layers with a softmax activation were added to normalize the prediction. For hyperparameters, Adam was used as an optimizer with learning rate = 1 × 10^–4^, and the training loss was set to binary cross entropy. Table S1 and Figure S2 show the metrics for the performance of this model.

We observed that the model could generalize well on the test data set. However, although an AUC score of 0.9 seemed appealing, there were many false positives in the predictions. This would be a critical issue when using this model as a reward function for reinforcement learning. The solution was to train another QSAR model that predicted molecular activity. The final reward function for reinforcement learning would then be based on the consensus of both models to increase the certainty of the prediction.

#### Reinforcement Learning Molecular Descriptors-Based Reward Function

We used chemical descriptors as predictors instead of SMILES string embeddings in the second QSAR model. The idea behind this approach was that chemical descriptors are generally better able to quantify molecular properties than SMILES^34^ and would reduce the classification problem and make it more explainable. Instead of learning molecular embeddings, the model would be learning measurable properties that could be compared among the molecules and associated with the output variable.

The chemical descriptors used as predictors were calculated by the PaDEL software. They were the 2D and 3D physicochemical properties of the molecules, such as molecular weight, ring count and the moment of inertia (1875 descriptors in total). This meant that there were more predictors than samples in the data set, indicating that the model would be unable to generalize and elevating the risk that the model would learn the noise (irrelevant features) in the data. The solution was to apply feature selection with genetic algorithms. Genetic algorithms are powerful in high-dimensional data sets with more features than samples because they can handle complex, nonlinear relationships between variables, whereas simple linear models such as Lasso rely on linear relationships.^36^ Genetic algorithms also do not assume any distribution for the data or the errors and they can be more effective in finding an optimal set of features as they use a heuristic search method to explore the feature space. One additional advantage of this approach was that it helped to identify the common chemical properties among the active molecules.

Hence, we applied a genetic algorithm to find the best-performing subset of features when training a RF model. The features considered for selection were the most important ones identified by the trained RF model, given its ability to rank features based on the impurity (Gini impurity) of its underlying decision trees.^37^ Feature importance values were calculated as the average of the impurity decrease accumulation within each decision tree of the model. A genetic algorithm mimics the process of natural selection to identify the subset of the most important features that maximize the model performance.^38^ First, an initial population of individuals was generated where each individual was a subset of features. The subsets were then scored by an RF model that predicted the target variable we were interested in the antiaggregation activity. Subsets with the highest scores were chosen to move to the next generation. Crossovers and mutations were applied so some features would switch places among the winner subsets while others would be added or removed randomly based on a mutation rate. Simple data cleaning and augmentation were applied before training the model and running the genetic algorithm to ensure data set balance.

**Figure 3:**
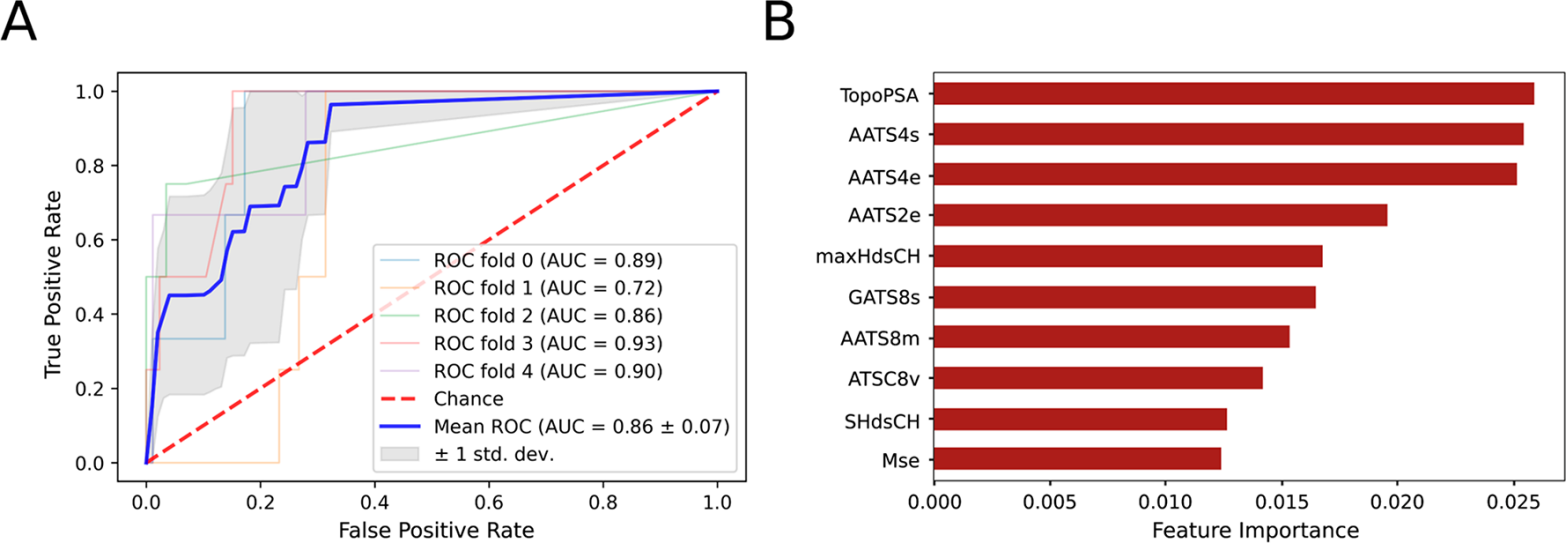
Metrics and important features in the descriptor-based RF QSAR model. (A) ROC AUC curve of the model with cross validation shown (AUC = 0.85). (B) Feature importance values derived from the RF QSAR model identify topological polar surface area as a key determinant.

A random grid search (with 3-fold cross-validation) was run for ten iterations to find the optimal hyperparameters for the RF model to ensure the best performance. After identification of the most important features, of which topological polar surface area was the most prominent, we ran the genetic algorithm to select the subset of features that maximized the classification performance. Figure 3 shows the ROC curve for this model and the features that were most strongly associated with the activity of the molecule according to the RFs. The hyperparameters used for RF and the genetic algorithm are shown in Tables S2 and S3.

The metrics for this model vs the SMILES embedding-based model are shown in Table 3. The descriptor-based model performance was an improvement, and it was better able to generalize than all previously trained models. The predictions had no false positives, and the model accuracy and average AUC scores were 0.98 and 0.85, respectively. We observed a considerable improvement in the metric macro averages in the descriptor-based model over the SMILES-based model, which meant higher classification scores for the positive class and fewer false negatives. On the other hand, there was not a large difference in the weighted average metrics, given that both models could classify inactive molecules efficiently. Hence, both models were used in the reinforcement learning reward function, but the descriptor-based classifier was given a higher weight, which was chosen based on the reinforcement learning performance.

**Table 3:** Comparison Table for Macro Average Metrics of the SMILES-Based Model vs Descriptor-Based Model, with Weighted Average Shown in Brackets^a^

	Metric		
Classifier	Precision	Recall	FI Score
SMILES based	0.74 (0.96)	0.74 (0.96)	0.74 (0.96)
Descriptor based	0.99 (0.98)	0.75 (0.98)	0.83 (0.97)

a Macro averages are shown with weighted averages in brackets.

#### Final Exploration Model

We trained a generative model for BBB-permeable molecules and defined 2 QSAR classifiers to filter the generated molecules based on antiaggregation potency. The overall model architecture was then fine-tuned using reinforcement learning, an extension of the GraphINVENT package. The agents learn how to optimize the APDs of the generative model in order to maximize the QSAR reward functions. The loss function used for training was the best agent reminder loss (BAR),^39^ which was responsible for the memory-awareness property of the model. This memorized the best agent with the highest score while training and was useful for reminding the new agents of the steps explored by previous agents to generate highly scoring molecules.

The fine-tuning process started by defining the prior and best agents and initializing them as the best performing MNN generative model outlined above. Then the following steps were repeated until the model converged to novel molecules with the highest scores:

- Generate a set of molecules using both priors (the current and the best).
- Score the molecules using the QSAR model.
- Compute the probabilities that the prior generative model and the current agent will assign the same actions carried out by the current agent to build a molecule.
- Compute the probabilities that the current and best agent will assign the same actions done by the best agent to build a molecule.
- Calculate the BAR loss and update the model weights to minimize it.

The prior generative model was the best performing MNN model outlined above. The hyperparameters were set as recommended in the initial paper, and the learning rate was set to 1 × 10^–4^. The best agent was updated every two epochs. The weights that were found to maximize the model performance after several training runs were 0.78 and 0.22 for the descriptor-based and SMILES-based models, respectively. The agents dealt with the score as a continuous value, meaning that the best agent was updated when the generated molecules gained a higher score than the last best score without any minimum thresholds for accepting the score.

After fine-tuning the model for 1000 epochs, it generated a set of novel small molecules that were predicted to be BBB permeable, druglike, and potentially able to delay the aggregation of αS. We observed that most molecules had a CNS MPO score higher than the threshold (0.9) as calculated by GuacaMol, which meant that they had a high probability of being able to cross the BBB.

#### Investigation of Generated Molecules and Experimental Testing of QSAR Models

**Figure 4:**
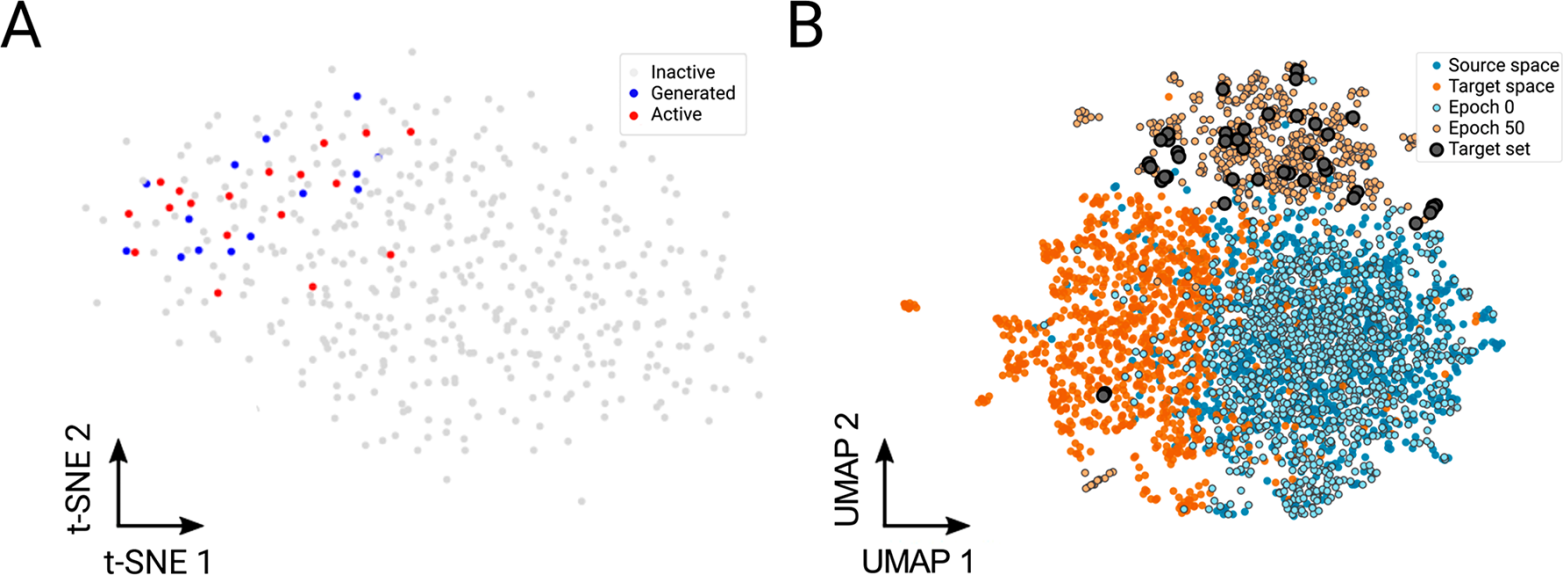
Chemical landscape of the exploration and exploitation pipelines. (A) Exploration pipeline. Comparison of the chemical space spanned by the chemical inhibitor training set and the newly generated compounds during the exploration strategy. t-SNE representation of the landscape of the chemical inhibitor training set with the original active (red) and nonactive (gray) compounds, and the newly generated compounds (blue). (B) Exploitation pipeline. UMAP representation of the CLM molecule generation process. With successive iterations the generated molecules take on features similar to the target set (previously identified aggregation inhibitors) while incorporating features of a target space (natural product library).

**Figure 5:**
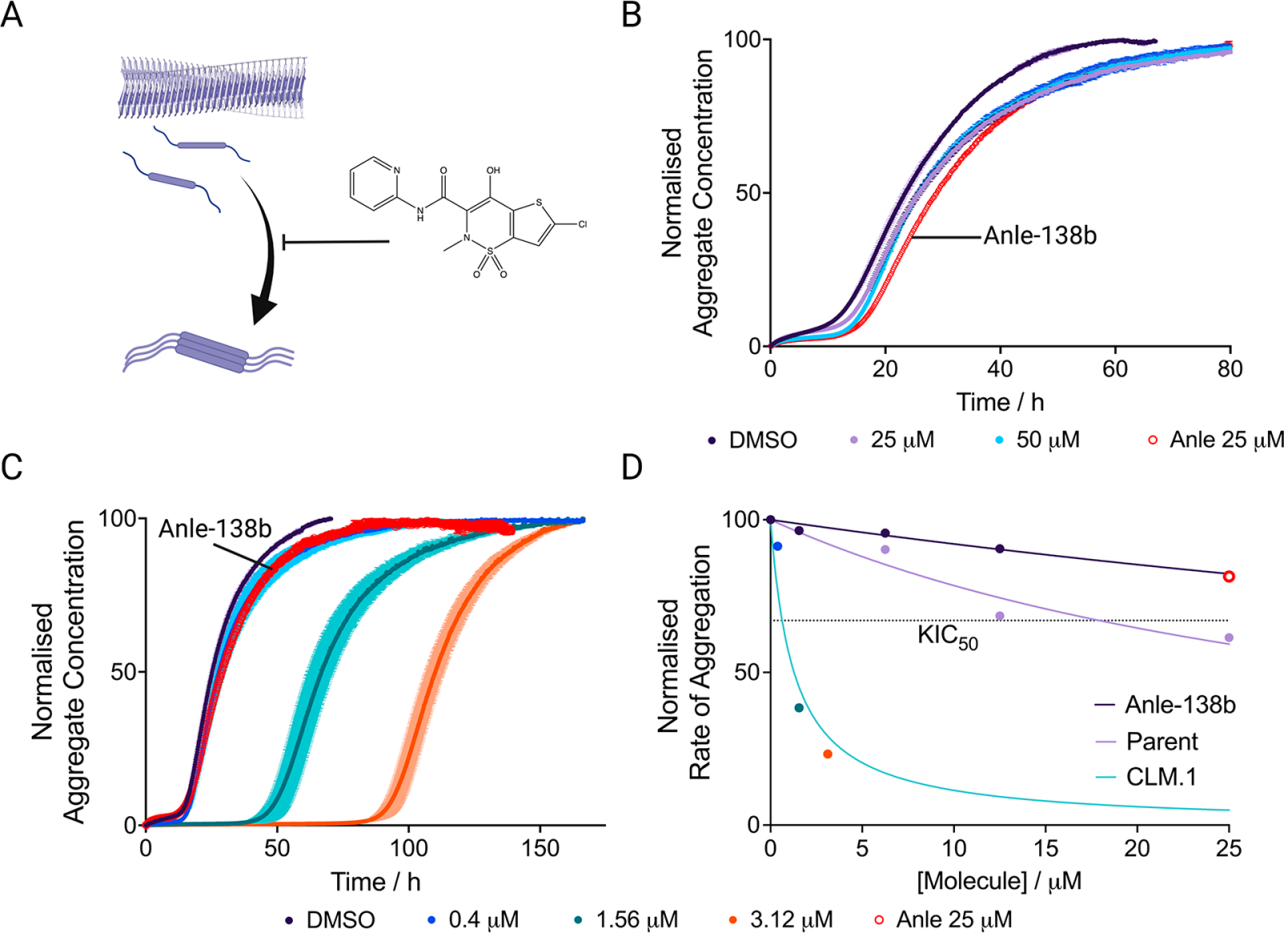
Experimental validation of compounds generated via the exploration and exploitation pipelines. (A) Schematic of the secondary nucleation process, which is the dominant mechanism in oligomer formation. Small molecules can block this process through a proposed mechanism^19^ of blocking fibril nucleation sites (lornoxicam is shown as an example). (B) Kinetic trace of a 10 μM solution of αS with 25 nM seeds at pH 4.8, 37 °C in the presence of lornoxicam at 25 μM (lilac) and 50 μM (light blue) or in the presence of 1% DMSO (dark purple). Anle-138b (red circles) at 25 μM is shown as a control. (C) Kinetic trace of a 10 μM solution of αS with 25 nM seeds at pH 4.8, 37 °C in the presence of CLM.1 at 0.4 μM (blue), 1.6 μM (teal) and 3.12 μM (orange), or in the presence of 1% DMSO (dark purple). Anle-138b (red circles) is shown as a control. The end points are normalized to the αS monomer concentration at the end of the experiment, which was detected via the Pierce BCA Protein Assay at *t* = 150 h. (D) Approximate rate of reaction (taken as 1/*t*_*1/2*_, normalized between 0 and 100) in the presence of 3 different molecules, Anle-138b (purple), the parent structure of CLM.1 (lilac) and CLM.1 (blue). The KIC_50_ of CLM.1 (0.42 μM) is indicated by the intersection of the fit and the horizontal dotted line.

While most of the molecules generated (Figure S3) were not obtainable without custom synthesis, they showed an overlap (according to tSNE^40^) in the chemical space with the active molecules in the chemical inhibitor data set (Figure 4A). As a test of whether the QSAR reward functions worked appropriately, we ordered a compound (lornoxicam) within the original training set with high predicted antiaggregation score to test experimentally in the aggregation assay used to generate the aggregation inhibition data set.^19^ This was a chemical kinetics assay,^9,41,42^ which identified the top compounds that significantly inhibit the surface-catalyzed secondary nucleation step in the aggregation of αS. This mechanism of action is relevant to disease since secondary nucleation is considered a significant mechanism in oligomer production.^4,8^ While this assay does not directly recapitulate the disease process, and it does not give a direct measure of oligomers, molecules previously screened through this approach showed both a prevention of aggregation seeded by diseased brain samples and also showed significant oligomer reduction.^19^ Therefore, the assay may act as a useful screening proxy to filter potential molecules before these challenging experiments are required for validation. The potency of lornoxicam was mild in comparison to hits found previously, but it was nonetheless observable and comparable to an antiaggregation compound in clinical development, Anle-138b (Figure 5). Since the original inhibitor training set only contained 4–6 distinctly different active structures, we anticipate that the performance of the model could improve as more varied training data are added, significantly reducing the resource cost of a potential exploration screening strategy.

### Exploitation Pipeline

The exploitation pipeline employed the CLM as previously described,^21^ using the bioactive library as a source space and the natural products library and the aggregation hits as the target space and target set, respectively. Over successive epochs, the generated molecules tended to assume more of the features of the target space and target set, with a greater weighting assigned to the latter. Applied to our data set, we employed a high number of training epochs to ensure the resultant molecules did not deviate too much from our selection of hit molecules, to increase the likelihood of potency. Initially we trialled different selections of compounds from the aggregation data set but found that using <30 epochs and including milder potency structures as the target set for the model would lead to a significant diversity of structures, but few that were likely to achieve potency. This architecture could also be used in an explorative approach by reducing the number of epochs and increasing the diversity of the target space, but with the limitation that different parameters such as potency and CNS MPO could not be explicitly optimized for and weighted, as in the GraphINVENT pipeline.

We used 50 training epochs and only the top 20 hit structures as the target set to ensure generated molecules were close in the chemical space to potent structures. A UMAP representation of this process is displayed in Figure 4B, which shows molecule generation in the proximity to the area of interest around the top 20 hits. Due to lack of availability of the generated compounds, a similarity search was carried out for the first 500 generated compounds at epoch 50, which were subsequently rescreened through the QSAR model developed previously. Twenty molecules were tested yielding 5 hits, 1 of which (labeled CLM.1) showed a greater level of novelty compared to previously identified structures, and exhibited high potency (Figure 5). The kinetic inhibitory constant (KIC_50_), the concentration of the molecule at which the *t*_*1/2*_ is increased by 50% with respect to the control as defined previously,^43^ was then derived. The KIC_50_ value of CLM.1 (0.42 μM) was an improvement on the best hit identified previously (KIC_50_ of 0.52 μM), both of which compare very favorably with the parent molecule of the original hit molecules and Anle-138b, which have extrapolated KIC_50_ values of 18.2 μM and 36.4 μM, respectively. The structures of the hits derived from the CLM strategy, and their respective normalized half times are shown in Figure S4.

## Conclusions

The objective of the machine learning approaches presented here was to demonstrate that small molecules balancing drug likeness, BBB penetrance, and aggregation inhibition could be predicted, providing useful tools for the therapeutic efforts against synucleinopathies. Our results illustrate the potential of generative machine learning methods to provide novel starting compounds with high likelihood of potency against αS aggregation. More generally, utilizing exploitation and exploration pipelines in series is an effective strategy that can be applied within research projects requiring improvements in performance of small molecules and biomolecules in various assays of interest, while retaining molecular properties integral to good target engagement. Key to the success in this approach is the tailoring of the architecture of the pipeline and the models within it for best performance, with greater emphasis placed on essential metrics. The pipelines that we have developed are concerned with the two main issues confronting research programs aimed at synucleinopathies: target engagement (in terms of BBB permeability) and potency (in terms of toxic oligomer reduction). The molecule tested for the exploration pipeline proved to be a mild inhibitor, but nonetheless offers a potential starting point for elaboration. Indeed, as shown previously,^19^ the potency of a hit compound can be improved upon many fold if an exploitation strategy is pursued. The exploitation strategy yielded a compound that exhibited smaller departures from the previous hit compounds and yielded high potency, while addressing the restrictive nature of the chemical space search approach previously employed. Overall, this we anticipate that approaches of the type described here will benefit researchers working in the field of protein misfolding diseases and drug discovery research in general.

## Materials and Methods

Full code can be found on the GitHub Repository: https://github.com/husseinmur/GraphINVENT-CNS.

### Compounds and Chemicals

Compounds were purchased from MolPort (Riga, Latvia) or Mcule (Budapest, Hungary) and prepared in DMSO to a stock of 5 mM. All chemicals used were purchased at the highest purity available.

### Recombinant αS Expression

Recombinant αS was purified based on previously described methods.^44−46^ The plasmid pT7-7 encoding for human αS was transformed into BL21 (DE3) competent cells. Following transformation, the competent cells were grown in 6L 2xYT media in the presence of ampicillin (100 μg/mL). Cells were induced with IPTG, grown overnight at 28 °C, and then harvested by centrifugation in a Beckman Avanti JXN-26 centrifuge with a JLA-8.1000 rotor at 5000 rpm (Beckman Coulter, Fullerton, CA). The cell pellet was resuspended in 10 mM Tris, pH 8.0, 1 mM EDTA, 1 mM PMSF, and lysed by sonication. The cell suspension was boiled for 20 min at 85 °C and centrifuged at 18,000 rpm with a JA-25.5 rotor (Beckman Coulter). Streptomycin sulfate was added to the supernatant to a final concentration of 10 mg/mL, and the mixture was stirred for 15 min at 4 °C. After centrifugation at 18,000 rpm, the supernatant was taken with an addition of 0.36 g/mL ammonium sulfate. The solution was stirred for 30 min at 4 °C and centrifuged again at 18,000 rpm. The pellet was resuspended in 25 mM Tris, pH 7.7, and the suspension was dialyzed overnight in the same buffer. Ion-exchange chromatography was then performed using a Q Sepharose HP column of buffer A (25 mM Tris, pH 7.7) and buffer B (25 mM Tris, pH 7.7, 1.5 M NaCl). The fractions containing αS were loaded onto a HiLoad 26/600 Superdex 75 pg Size Exclusion Chromatography column, and the protein (≈60 mL @ 200 μM) was eluted into the required buffer. The protein concentration was determined spectrophotometrically using ε275 = 5600 M^–1^ cm^–1^.

### Seed Fibril Preparation

αS fibril seeds were produced as described previously.^44,45^ Samples of αS (700 μM) were incubated in 20 mM phosphate buffer (pH 6.5) for 72 h at 40 °C and stirred at 1,500 rpm with a Teflon bar on an RCT Basic Heat Plate (IKA, Staufen, Germany). Fibrils were then diluted to 200 μM, aliquoted and flash frozen in liquid N_2_, and finally stored at −80 °C. For the use of kinetic experiments, the 200 μM fibril stock was thawed, and sonicated for 15 s using a tip sonicator (Bandelin, Sonopuls HD 2070, Berlin, Germany), using 10% maximum power and a 50% cycle.

### Measurement of Aggregation Kinetics

αS was injected into a Superdex 75 10/300 GL column (GE Healthcare) at a flow rate of 0.5 mL/min and eluted in a 20 mM sodium phosphate buffer (pH 4.8) supplemented with 1 mM EDTA. The obtained monomer was diluted in buffer to a desired concentration and supplemented with 50 μM ThT and preformed αS fibril seeds. The molecules (or DMSO alone) were then added at the desired concentration to a final DMSO concentration of 1% (v/v). Samples were prepared in low-binding Eppendorf tubes, and then pipetted into a 96-well half area, black/clear flat bottom polystyrene NBS microplate (Corning 3881), 150 μL per well. The assay was then initiated by placing the microplate at 37 °C under quiescent conditions in a plate reader (FLUOstar Omega, BMG Labtech, Aylesbury, UK). The ThT fluorescence was measured through the bottom of the plate with a 440 nm excitation filter and a 480 nm emission filter. After centrifugation at 5000 rpm to remove aggregates, the monomer concentration was measured via the Pierce BCA Protein Assay Kit according to the manufacturer’s protocol.

## Data Availability

Full code can be found on the GitHub Repository: https://github.com/husseinmur/GraphINVENT-CNS.

## Supporting Information Available

Model performance metrics and hyperparameter values not mentioned in the main text. The referenced supplementary figures can also be found here, providing information on data distributions of the molecule data sets, ROC curves for initially trialled models, molecule structures generated for the different pipelines and αS aggregation data in the presence of inhibitors (PDF)
