# Supplementary material for: Exploration and Exploitation Approaches Based on Generative Machine Learning to Identify Potent Small Molecule Inhibitors of α-Synuclein Secondary Nucleation
Source: J Chem Theory Comput. 2023 Mar 20;19(14):4701–10. doi: 10.1021/acs.jctc.2c01303 (PMC10373478; doi:10.1021/acs.jctc.2c01303)
Supplement: Supplementary file 1 — ct2c01303_si_001.pdf [file ct2c01303_si_001.pdf]

SUPPORTING INFORMATION

Exploration and Exploitation Approaches Based on Generative Machine Learning to Identify Potent Small Molecule Inhibitors of α-Synuclein Secondary Nucleation

Robert I. Horne[1], Mhd Hussein Murtada[1], Donghui Huo[1,2], Z. Faidon Brotzakis[1], Rebecca C. Gregory[1], Andrea Possenti[1], Sean Chia[1,3], Michele Vendruscolo[1+]

[1]Centre for Misfolding Diseases, Yusuf Hamied Department of Chemistry, University of Cambridge, Cambridge CB2 1EW, UK

[2]College of Life Science and Technology, Beijing University of Chemical Technology, Beijing 100029, China

*[3]Bioprocessing Technology Institute, Agency of Science, Technology and Research (A*STAR), Singapore 138668, Singapore*

[+] Correspondence to: mv245@cam.ac.uk

Keywords

Parkinson’s disease; α-synuclein; protein misfolding; machine learning; generative modelling; multi parameter optimisation; kinetic-based drug discovery

SUPPORTING INFORMATION

Average	Metric		
	Precision	Recall	F1 Score
Micro Average	0.96	0.96	0.96
Macro Average	0.74	0.74	0.74
Weighted Average	0.96	0.96	0.96

Table S1. Metrics for SMILES embedding based model performance on aggregation data.

Number of estimators	1800
Minimum samples split	5
Minimum samples at a leaf node	1
Maximum depth	70
Bootstrap	False

Table S2: RF parameters used during genetic algorithm selection.

Maximum number of features in subset	5
No. of individuals is starting population	100
Probability of crossover	0.5
Probability of mutation	0.2
Number of generations	50

Table S3: Genetic algorithm parameters.

SUPPORTING INFORMATION

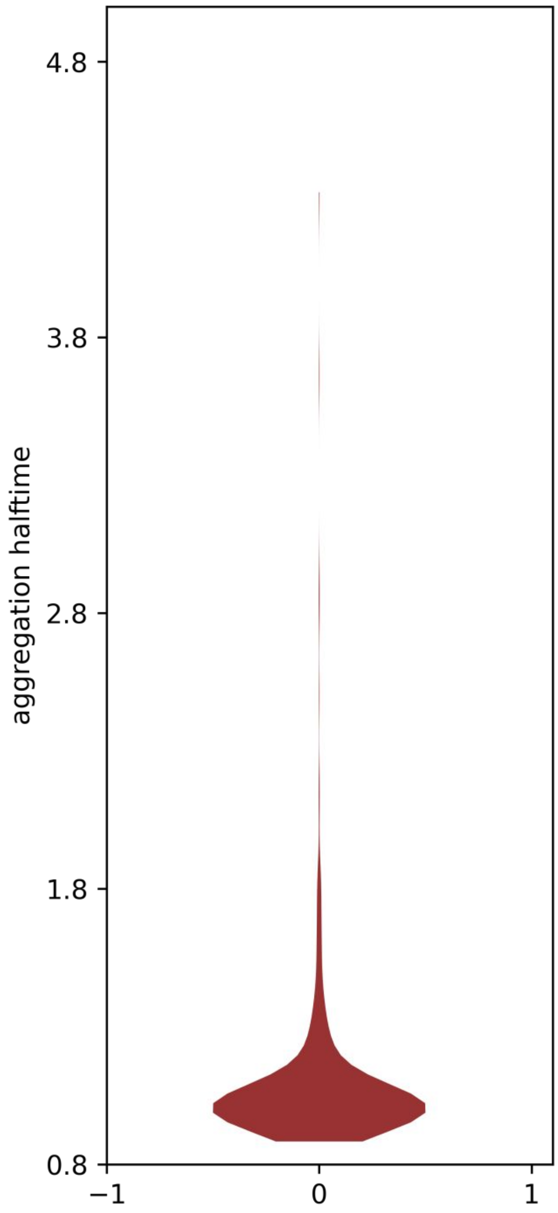

Figure S1. Distribution of normalised aggregation half times in the αS aggregation inhibitor data set. The dataset of known aggregation inhibitors was unbalanced towards having many more inactive than active compounds.

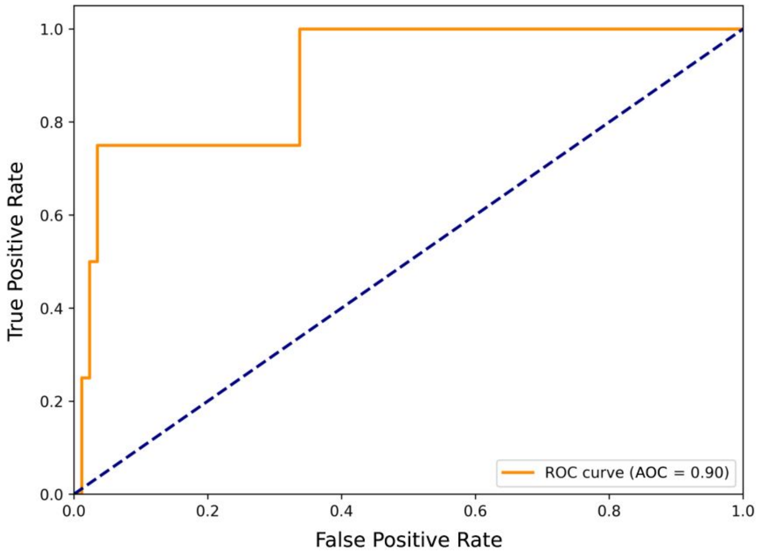

Figure S2. ROC AUC curve for the SMILES embedding model initially appeared promising with an AUC of 0.9.

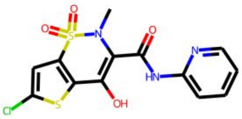

CNS MPO: 0.91

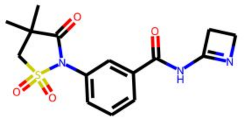

CNS MPO: 0.97

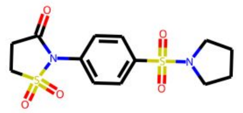

CNS MPO: 1.0

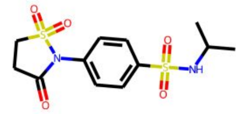

CNS MPO: 0.96

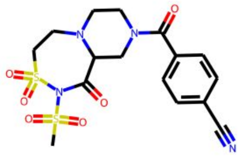

CNS MPO: 0.77

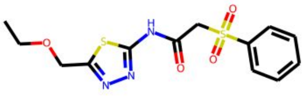

CNS MPO: 0.97

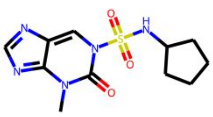

CNS MPO: 0.97

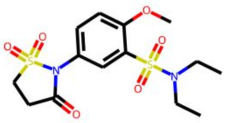

CNS MPO: 0.98

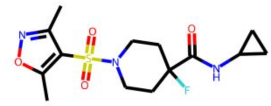

CNS MPO: 0.98

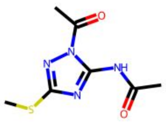

CNS MPO: 0.98

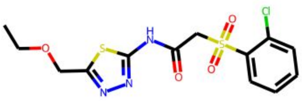

CNS MPO: 0.96

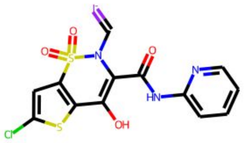

CNS MPO: 0.73

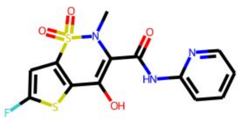

CNS MPO: 0.91

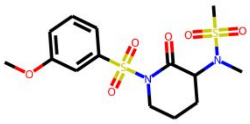

CNS MPO: 0.98

Figure S3. Structures generated by the final pipeline and their respective calculated CNS MPO scores.

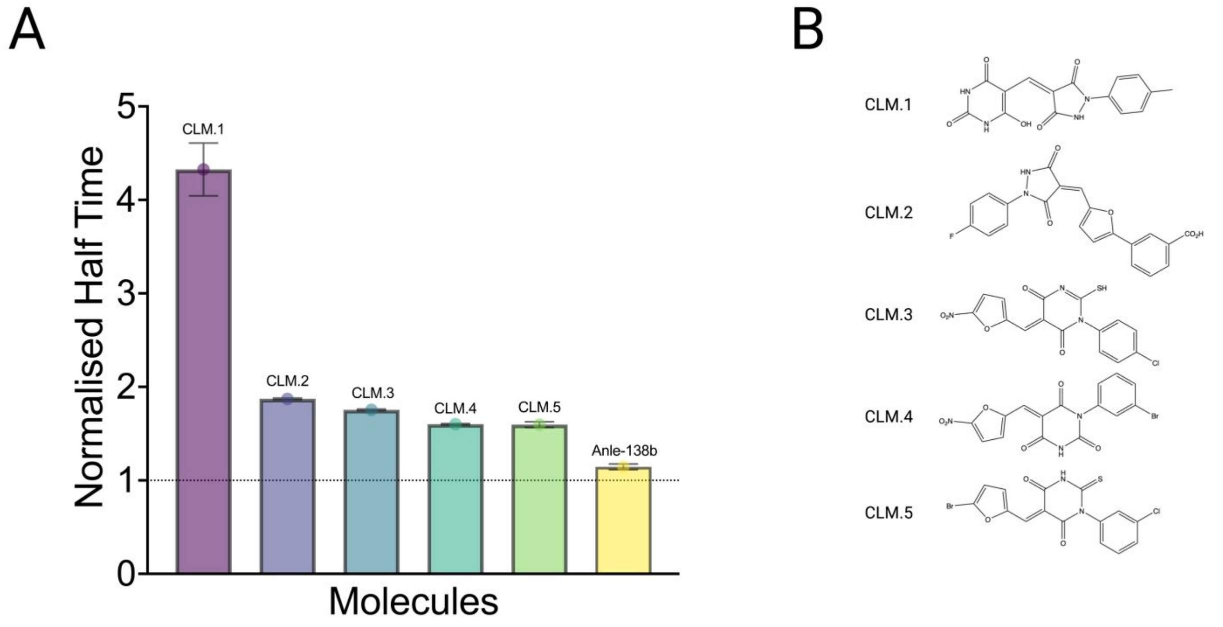

Figure S4. Aggregation data from the new hits generated via the exploitation pipeline. (**A**) Normalised half times for a 10 µM solution of αS with 25 nM seeds at pH 4.8, 37 °C in the presence of CLM generated molecules at 3.12 µM. The horizontal dotted line indicates the normalised half time of a 1% DMSO negative control. Anle-138b at 25 µM is also shown for comparison. (**B**) Structures of the CLM generated molecules.
